# Production of phenylacetyl-homoserine lactone analogs by artificial biosynthetic pathway in *Escherichia coli*

**DOI:** 10.1186/s12934-015-0379-1

**Published:** 2015-11-25

**Authors:** Sun-Young Kang, Jae Kyoung Lee, Jae-Hyuk Jang, Bang Yeon Hwang, Young-Soo Hong

**Affiliations:** Chemical Biology Research Center, Korea Research Institute of Bioscience and Biotechnology, 30 Yeongudanji-ro, Ochang-eup, Chungbuk, 363-883 Republic of Korea; Department of Pharmacy Graduate School, Chungbuk National University, Cheongju, 361-763 Republic of Korea

**Keywords:** Homoserine lactone (HSL), Phenylacetyl-HSL, *p*-coumaroyl-HSL, Artificial biosynthesis

## Abstract

**Background:**

Quorum sensing (QS) networks are more commonly known as acyl homoserine lactone (HSL) networks. Recently, *p*-coumaroyl-HSL has been found in a photosynthetic bacterium. *p*-coumaroyl-HSL is derived from a lignin monomer, *p*-coumaric acid, rather than a fatty acyl group. The *p*-coumaroyl-HSL may serve an ecological role in diverse QS pathways between *p*-coumaroyl-HSL producing bacteria and specific plants. Interference with QS has been regarded as a novel way to control bacterial infections. Heterologous production of the QS molecule, *p*-coumaroyl-HSL, could provide a sustainable and controlled means for its large-scale production, in contrast to the restricted feedback regulation and extremely low productivity of natural producers.

**Results:**

We developed an artificial biosynthetic process for phenylacetyl-homoserine lactone analogs, including cinnamoyl-HSL, *p*-coumaroyl-HSL, caffeoyl-HSL, and feruloyl-HSL, using a bioconversion method via *E. coli* (CB1) in the co-expression of the codon-optimized LuxI-type synthase (RpaI) and *p*-coumaroyl-CoA ligase (4CL2nt). In addition to this, we show the de novo production of *p*-coumaroyl-HSL in heterologous host *E. coli* (DN1) and tyrosine overproducing *E. coli* (DN2), containing the *rpaI* gene in addition to *p*-coumaroyl-CoA biosynthetic genes. The yields for *p*-coumaroyl-HSL reached 93.4 ± 0.6 and 142.5 ± 1.0 mg/L in the S-adenosyl-l-methionine and l-methionine feeding culture in the DN2 strain, respectively.

**Conclusions:**

This is the first report of a de novo biosynthesis in a heterologous host yielding a QS molecule, *p*-coumaroyl-HSL from a glucose medium using a single vector system combining *p*-coumaroyl-CoA biosynthetic genes and the LuxI-type synthase gene.

**Electronic supplementary material:**

The online version of this article (doi:10.1186/s12934-015-0379-1) contains supplementary material, which is available to authorized users.

## Background

Bacteria use small molecules and peptides to assess their local population densities in a process termed quorum sensing (QS) [1, 2]. Quorum sensing is a regulatory system used by bacteria for controlling gene expression in response to increasing cell density [3, 4]. When bacteria reach a sufficiently high population density, they will alter gene expression so as to carry out a range of processes that require the cooperation of a large number of cells [5]. These regulatory processes are remarkable in their diversity, ranging from virulence factor and antibiotic production to biofilm formation, root nodulation, and bioluminescence, and have direct and often devastating impacts on the bacterial host [6]. Many bacteria use acyl-HSL synthases to generate fatty acyl-HSL quorum-sensing signals, which function with signal receptors to control the expression of specific genes. The fatty acyl group is derived from fatty acid biosynthesis and provides signal specificity, but the variety of signals is limited [2].

Meanwhile, the photosynthetic bacterium *Rhodopseudomonas palustris* produces a natural phenylacetyl-HSL, *p*-coumaroyl-HSL, using a LuxI-type synthase (RpaI, also known as *p*-coumaryl-homoserine lactone synthase, EC 2.3.1.229) with a *p*-coumaric acid rather than fatty acids [7, 8]. Schaefer et al. speculated that there is an intimate relationship between *p*-coumaroyl-HSL producing bacteria and specific plants through *p*-coumaroyl-HSL signaling [7], because the *rpaI* gene expression is activated specifically by growth of *R. palustris* on *p*-coumaric acid [9], a major aromatic monomer of lignin, which comprise over 30 % of all plant dry material. It is clear that several photosynthetic bacteria and nitrogen-fixing bacteria respond in complex ways to the presence of exogenous lignin monomers and this may drive intercellular signaling for a metabolites’ production [7, 10]. These metabolites may comprise antibiotics and auxins that suppress the growth of potentially parasitic bacteria and promote algal growth, respectively [11–13]. It is a distinctly possible scenario that phenylacetyl-HSL could serve an ecological role in these diverse QS pathways in natural environments.

Recently there have been intensive efforts by several groups to find small molecules that can interrupt the QS communication among bacteria [12, 14–20]. It is believed that disrupting bacterial communication and hence virulence factor production would not put substantial evolutionary pressure on bacteria to develop resistance. To address these challenges, non-native synthetic phenyl or phenol HSL analogs have shown significant activity against TraR, a QS receptor, in *Agrobacterium tumefaciens* and were 1–2 orders of magnitude more active than the previously reported LuxR-type protein antagonists examined as controls. Impressively, bromophenyl-HSL displayed 50 % inhibition at an equimolar concentration of natural fatty acyl-HSL, N-3-oxooctanoyl-HSL [15–17, 20]. Interestingly, a close relative of *p*-coumaroyl-HSL, cinnamoyl-HSL, was shown to have limited activity against reporter strains for traditional HSLs [16]. These phenylacetyl-HSL compounds highlight the potential for autoinducer libraries with substantial structural diversity, to serve as probes or modulators for QS circuits mediated by ‘nonstandard’ signals [21].

Homoserine lactone biosynthesis typically involves a series of reactions that use S-adenosyl methionine (SAM) as the amino donor to generate the HSL ring moiety, and fatty-acyl carrier protein (ACP) or -acyl coenzyme A (CoA) as the precursor for the N-acyl side chain of the HSL molecules [11, 22]. When grown in the presence of *p*-coumaric acid, a few bacteria, *R. palustris*, *Bradyrhizobium* sp. BTAi1 and *Silicibacter pomeroyi* DSS-3, produced relatively small amounts (10 μM) of *p*-coumaroyl-HSL [7]. In this study, we tested the substrate specificity of RpaI towards phenolic acids CoA e.g., cinnamoyl-, *p*-coumaroyl-, caffeoyl-, and feruloyl-CoA. In addition to this, we show the *de novo* production of *p*-coumaroyl-HSL in heterologous host *E. coli* containing an artificial biosynthetic pathway that contained the *rpaI* gene in addition to *p*-coumaroyl-CoA biosynthetic genes. The production of *p*-coumaroyl-HSL was about three fold higher in the engineered tyrosine overproducing *E. coli* strain compared to that of the wild type *E. coli.* Finally, the yields for *p*-coumaroyl-HSL were 93.4 ± 0.6 and 142.5 ± 1.0 mg/L, respectively, by the tyrosine overproducing *E. coli* with SAM, or the l-methionine feeding strategy.

## Results and discussion

### In vitro enzymatic synthesis of phenolic acids to phenylacetyl-homoserine lactone analogs

**Fig. 1:**
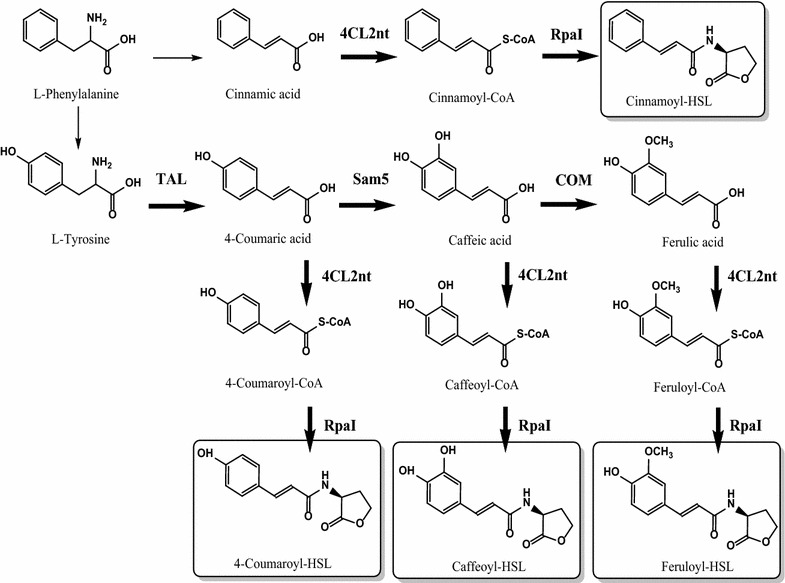
Engineered biosynthetic pathways for the phenylacetyl-HSL analogs in *E. coli*. The acyl-HSL synthase RpaI from *Rhodopseudomonas palustris* catalyzes the conversion of phenolic acid coenzyme A to phenylacetyl-HSL. The phenolic acid substrate is achieved through the conversion of l-tyrosine to *p*-coumaric acid by a tyrosine ammonia lyase (TAL) from *Saccharothrix espanaensis*, hydroxylation of *p*-coumaric acid to caffeic acid by Sam5 from *S.* *espanaensis*, and *O*-methylation of caffeic acid to ferulic acid by *O*-methyltransferase (COM) from *Arabidopsis thaliana*. A 4-coumarate-CoA ligase from *Nicotiana tabacum* (4CL2nt) generates the CoA-ester of the phenolic acid substrates, allowing lactone formation catalyzed by RpaI

**Fig. 2:**
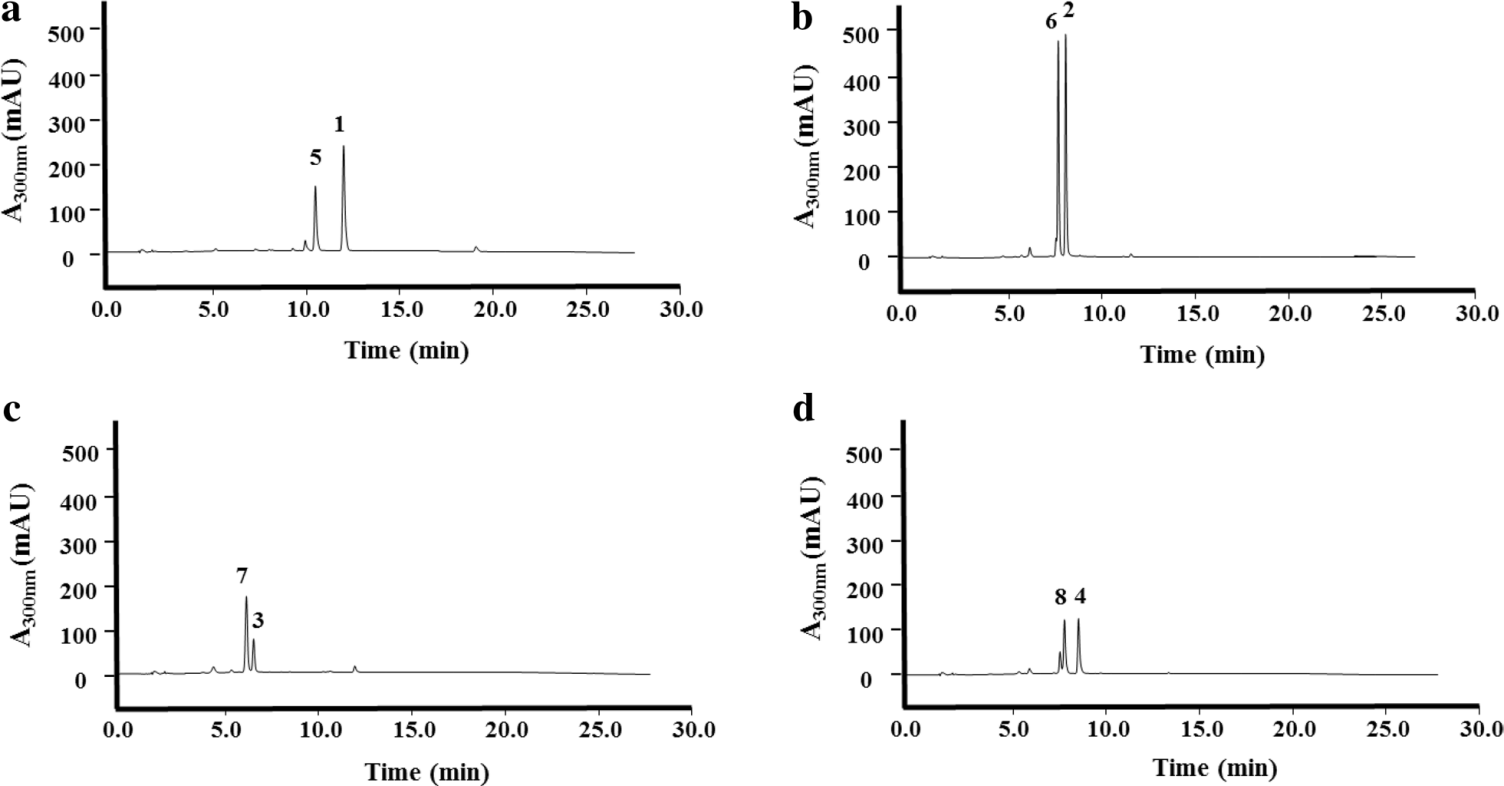
HPLC analysis of the in vitro enzymatic reaction with *p*-coumaric acid CoA ligase (4CL2nt) and acyl-HSL synthase (RpaI). The detection wavelength was 300 nm for reactions with cinnamic acid (**a**), *p*-coumaric acid (**b**), caffeic acid (**c**) and ferulic acid (**d**). *Peak 1*, cinnamic acid; *peak 2*, *p*-coumaric acid; *peak 3*, caffeic acid; *peak 4*, ferulic acid; *peak 5*, cinnamoyl-HSL; *peak 6*, *p*-coumaroyl-HSL; *peak 7*, caffeoyl-HSL; *peak 8*, feruloyl-HSL

RpaI, a LuxI-type synthase, was demonstrated to be a *p*-coumaroyl-HSL synthase [7]. On the basis of this precedent, we attempted enzymatic production using the purified His-tagged RpaI with various phenolic acids-CoA, in order to make novel phenylacetyl-HSL analogs (Fig. 1). In order to complete an initial survey of the substrate specificity of the RpaI enzyme, as a start the *p*-coumaric acid-CoA ligase (4CL2nt, EC 6.2.1.12) and four phenolic acids (cinnamic acid, *p*-coumaric acid, caffeic acid, and ferulic acid) were used in our in vitro enzyme reaction. The 4CL2nt from *Nicotiana tabacum* had already been identified with a broad substrate specificity for cinnamic acid, *p*-coumaric acid, caffeic acid, and ferulic acid [23]. The reaction of each phenolic acid with 4CL2nt enzyme in the presence of RpaI led to the formation of a new product, which was detected by HPLC (Fig. 2).

The putative phenylacetyl- HSL analogs appeared as a UV spectrum pattern similar to *p*-coumaroyl-HSL (broad peak of maximum absorbance at 308 nm and a minor peak at 227 nm) in the HPLC analyses (Additional file 1: Figure S1). These compounds were further analyzed using liquid chromatography-mass spectrometry (LC–MS) (Additional file 1: Figure S2). The peak at *m*/*z* 248 [M+H]^+^, which corresponded to *p*-coumaroyl-HSL, was detected in significant amounts with *p*-coumaric acid as a precursor. It was expected that the *p*-coumaric acid converted to *p*-coumaroyl-HSL through lactonization reaction of the *p*-coumaroyl-CoA.

**Fig. 3:**
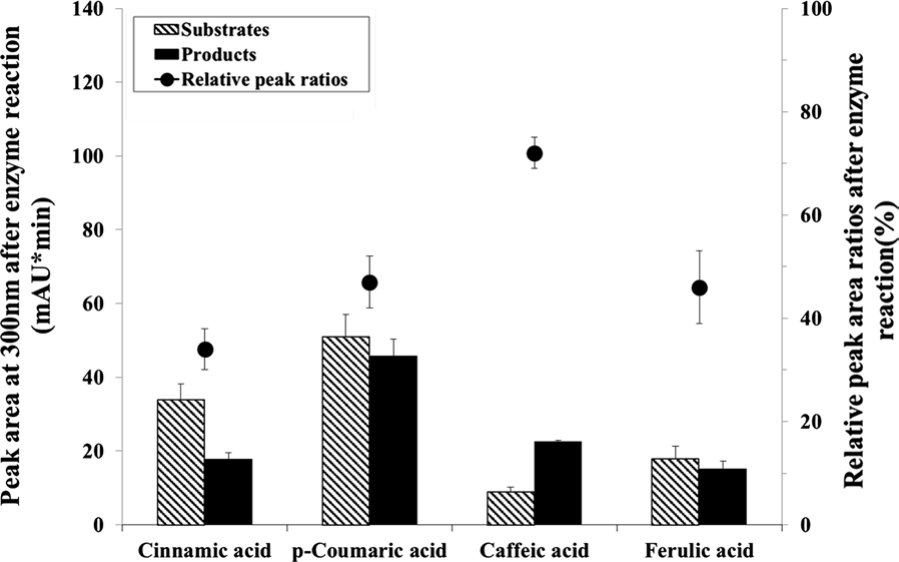
Relative HPLC peak area ratio between each product and substrate after enzymatic reactions. The HPLC peak area of remaining substrates (*lines*) and acyl-HSL products (*black*) were analyzed after each enzymatic reaction with cinnamic acid, *p*-coumaric acid, caffeic acid or ferulic acid. The ratios (*circles*) were calculated by peak area of each product against each remaining substrate at the end of enzymatic reactions. The detection wavelength was 300 nm for reactions with each phenolic acid. *Error bars* reported at one standard deviation from triplicate experiments

In addition, we found three putative HSL peaks in LC/MS analyses using cinnamic acid, caffeic acid, and ferulic acid as each starter substrate; each peak showed a molecular ion at *m*/*z* 232, *m*/*z* 264, and *m*/*z* 278 [M+H]^+^, respectively (Additional file 1: Figure S2). A full scan and MS^2^ mass spectral data for this putative lactone product showed a loss of 102 Da (HSL moiety) from the parent ion, which is a distinguishing pattern of the phenylacetyl-HSL analogs. When the relative HPLC peak area was calculated based on a quantitative comparison with the substrates and the products after reactions, roughly 34, 47, 72, and 46 % conversion ratios were shown for cinnamic acid, *p*-coumaric acid, caffeic acid, and ferulic acid, respectively (Fig. 3). Interestingly, the relative conversion ratio with caffeic acid is higher than the ratio of *p*-coumaric acid, which is the previously reported precursor of the RpaI enzyme [7]. In addition, the 4CL2nt had already confirmed that the relative activities toward caffeic acid were 25 %, compared to *p*-coumaric acid as substrates [23]. Taking these results together, we considered the possible explanation that the caffeoyl-CoA was a more suitable substrate for the RpaI enzyme. But, we found it difficult to analyze the RpaI enzyme kinetic data to obtain valid substrate specificity, because the caffeoyl-CoA compound was not commercially available.

### Bioconversion of phenolic acids to phenylacetyl-homoserine lactone analogs in *E. coli*

**Fig. 4:**
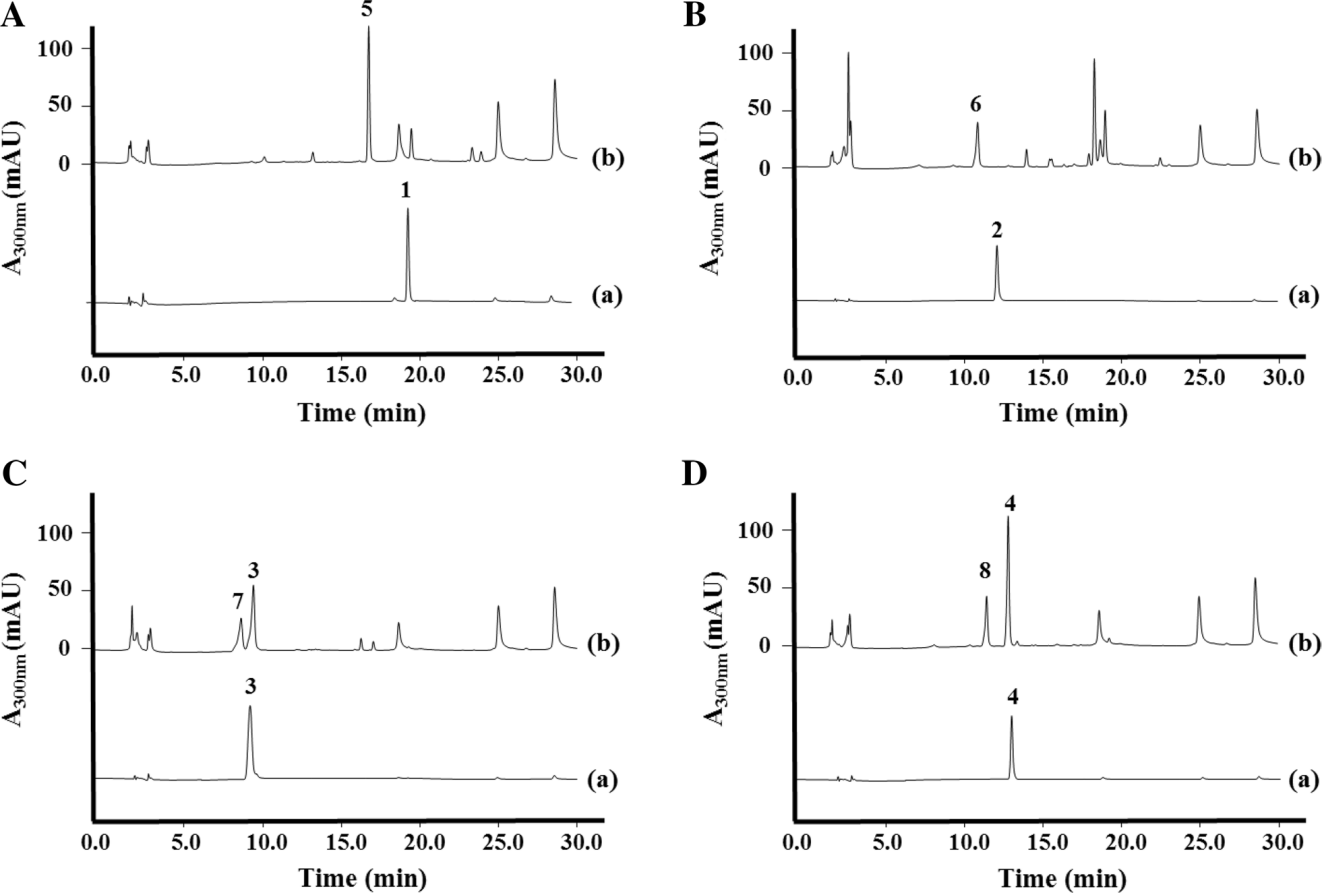
Bioconversion experiments with each phenolic acid. **A** HPLC profile of the standard cinnamic acid (*a*) and cinnamic acid supplemented *E. coli* C41 (DE3) harboring pET-4R (CB1) (*b*); **B** standard *p*-coumaric acid (*a*) and *p*-coumaric acid supplemented *E. coli* harboring pET-4R (CB1) (*b*); **C** standard caffeic acid (*a*) and caffeic acid supplemented CB1 strain (*b*); **D** standard ferulic acid (*a*) and ferulic acid supplemented CB1 strain (*b*). *Peak 1*, cinnamic acid; *peak 2*, *p*-coumaric acid; *peak 3*, caffeic acid; *peak 4*, ferulic acid; *peak 5*, cinnamoyl-HSL; *peak 6*, *p*-coumaroyl-HSL; *peak 7*, caffeoyl-HSL; *peak 8*, feruloyl-HSL

In order to more effectively obtain an amount of the novel phenylacetyl-HSL analogs, we constructed a bioconversion system for production of the novel phenylacetyl-HSL analogs utilizing the codon-optimized *p*-coumaroyl-HSL synthase gene (*rpaI*) addition with *p*-coumaroyl-CoA ligase gene (*4cl2nt*). The synthetic *rpaI* and *4cl2nt* genes were cloned into the expression vector pET-28a(+) using previously described cloning methods [24, 25], which resulted in pET-4R (Table 1). The four phenolic acids were added to the cultured recombinant *E. coli* C41(DE3) strain (CB1) with the *rpaI* and *4cl2nt* genes (pET-4R). The CB1 culture broth and bacterial cells were collected after 24 h culture and were then subjected to HPLC analyses (Fig. 4). Under the bioconversion condition employed in this study, cinnamic acid, *p*-coumaric acid, caffeic acid, and ferulic acid were each converted to phenylacetyl-HSL, respectively.

**Table 1:** Plasmids and strains used in this study

Plasmid or strain	Relevant characteristics	References
Plasmid		
pET-28a(+)	f1 ori, T7 promoter, Kan^R^	Novagen
pET-opTAL	pET-28a(+) carrying codon-optimized tyrosine ammonia lyase gene (*tal*)	Kang et al. [30]
pET-4CL2nt	pET-28a(+) carrying codon-optimized *p*-coumaroyl CoA ligase *4CL2* gene (*4cl2nt*)	This study
pET-opRpaI	pET-28a(+) carrying codon-optimized homoserine synthase gene (*rpaI*)	This study
pET-4R	pET-28a(+) carrying codon-optimized *4cl2nt* and *rpaI*	This study
pET-opT4R	pET-28a(+) carrying codon-optimized *tal*, *4cl2nt* and *rpaI*	This study
Strain		
*E. coli* DH5a	Cloning host	Invitrogen
*E. coli* C41(DE3)	Derivative strain of *E. coli* BL21(DE3)	Miroux and Walker [31]
ΔCOS1	*E. coli* C41(DE3); Δ*tyrR*::*tyrA* ^fbr^, *aroG* ^fbr^; tyrosine overproducing strain	Kang et al. [27]
CB1	*E. coli* C41(DE3) harboring pET-4R	This study
DN1	*E. coli* C41(DE3) harboring pET-opT4R	This study
DN2	*E. coli* ΔCOS1 harboring pET-opT4R	This study

To obtain NMR-accessible amounts from the present bioconversion conditions, 20 mg of each phenolic acid were added to a 2 L fermentation of CB1 strain. The structures of the purified phenylacetyl-HSL analogs were identified through spectral data interpretation and compared with the values reported in the literature [7]. The presence of HSL ring moieties were revealed by the ^1^H NMR data [*δ*_H_ 4.68–4.70 (1H, H-10), 4.24–4.39 (2H, H-12), and 2.21–2.48 (2H, H-13)] for all compounds. Additionally, three exchangeable proton signals were observed in DMSO-*d*_6_, at *δ*_H_ 9.42 (1H, 4-OH), 9.16 (1H, 3-OH), and 8.52 (1H, -NH) which were assigned as two hydroxyl protons at the benzene ring and amide proton of caffeoyl-HSL. And also, feruloyl-HSL showed one methoxyl group at *δ*_H_ 3.81 (3H, 3-OCH) and one hydroxyl group at *δ*_H_ 9.49 (1H, 4-OH) on the benzene ring. In the ^1^H NMR spectra, the large coupling constant (*J*_7,8_ = 15.7 Hz) implied the _*E*_-olefin relationship between C-7 and C-8 at the aryl side chain of each phenylacetyl-HSL compounds (Additional file 1: Tables S1, S2).

### Construction of de novo artificial biosynthetic pathways in *E. coli* to produce *p*-coumaroyl-HSL

In addition, although the production of *p*-coumaroyl-HSL has been established by the above enzymatic reaction and bioconversion, we also describe a different approach for their de novo synthesis in *E. coli* by engineering an artificial biosynthetic pathway. This could be a useful approach for economic production by one-pot fermentation without a precursor feeding process. We constructed the artificial de novo biosynthesis pathway for production of *p*-coumaroyl-HSL utilizing the *rpaI* gene addition with *p*-coumaroyl-CoA biosynthetic genes. The *p*-coumaroyl-CoA biosynthetic pathway genes are the tyrosine ammonia lyase gene (*tal*) and *p*-coumaroyl-CoA ligase gene (*4cl2nt*), which converts tyrosine to *p*-coumaroyl-CoA through the *p*-coumaric acid. Tyrosine ammonia lyases (TAL, EC 4.3.1.25) identified from various sources can catalyze the direct formation of *p*-coumaric acid from tyrosine. We have already succeeded in synthesizing *p*-coumaric acid in *E. coli* from a simple medium without the addition of tyrosine using TAL from *Saccharothrix espanaensis* [24].

**Fig. 5:**
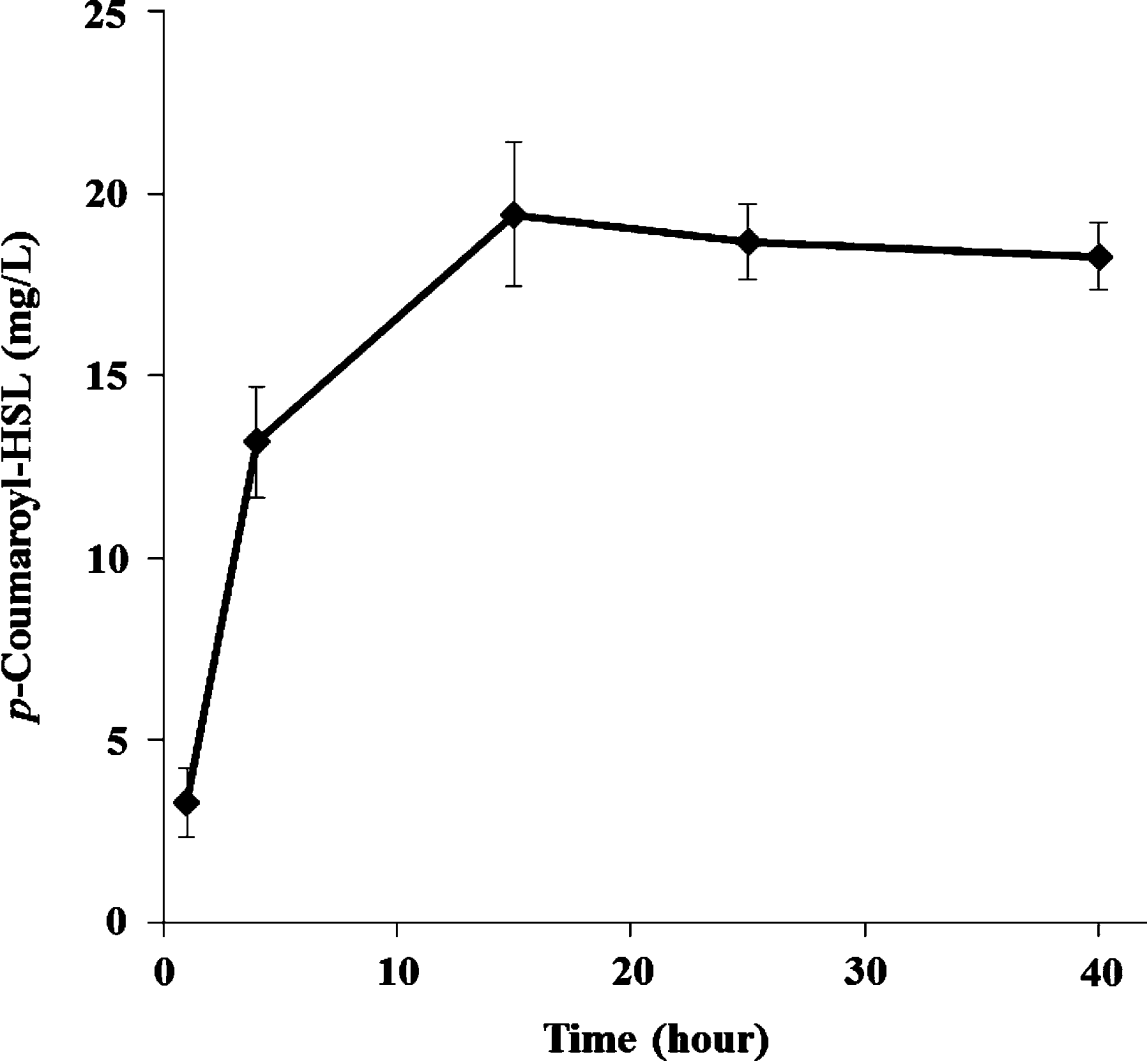
De novo biosynthesis of *p*-coumaroyl-HSL by DN1 strain. *p*-Coumaroyl-HSL production from glucose using *E. coli* C41(DE3) harboring pET-opT4R (DN1) during 40 h culture. *Error bars* reported at one standard deviation from triplicate experiments

For the de novo synthesis of *p*-coumaroyl-HSL in *E. coli*, the only requirement was to replace the *rpaI* gene in the previously described plant polyketide expression vector (Additional file 1: Figure S3) [24–26]. This method is one of the advantages of assembling a biosynthetic pathway for a certain product; replacing a single enzyme gives a different product, the structure of which depends on its catalytic properties. The final pET-opT4R vector contains the tyrosine ammonia lyase, *p*-coumaroyl-CoA ligase, and *p*-coumaroyl-HSL synthase genes. The recombinant strain (DN1) that harbors the artificial biosynthetic gene cluster (pET-opT4R) was cultured in a modified synthetic medium (SM) [27]. The *p*-coumaroyl-HSL peak was detected in the culture broth of the DN1 strain by HPLC. Additionally, the DN1 strain was investigated using metabolite pattern analyses based on the culture times, until the production of *p*-coumaroyl-HSL was saturated after 15 h. The amount of *p*-coumaroyl-HSL reached 19.4 ± 2.0 mg/L (78.5 μM) at 15 h (Fig. 5). This productivity shows a remarkable improvement over the titers (~10 μM) of the original producer, a *R. palustris* [7].

### Improved production of *p*-coumaroyl-HSL in a tyrosine overproducing *E. coli* strain

*p*-Coumaric acid is the pivotal intermediate of the plant phenylpropanoid pathway starting from the deamination of tyrosine. Thus tyrosine serves as an immediate endogenous precursor to the *p*-coumaroyl-HSL biosynthesis pathway. Recently, we reported engineered l-tyrosine overproducing *E. coli* ΔCOS1 strains via a deregulating of the aromatic amino acid biosynthesis pathway [27]. The tyrosine producer, *E. coli* ΔCOS1, was engineered on the genome to overexpress the feedback inhibition resistant (fbr) derivative genes of 3-deoxy-d-arabinoheptulosonate-7-phosphate synthase (*aroG*^*fbr*^) and chorismate mutase (*tyrA*^*fbr*^) in the repressor gene (*tyrR*) deletion strain. The tyrosine-overproducing strains showed a substantial capacity for *p*-coumaric acid, caffeic acid and ferulic acid biosynthesis [27]. Therefore, it is a suitable platform strain for the production of other tyrosine-derived aromatic compounds, using the phenolic acids as precursors.

**Fig. 6:**
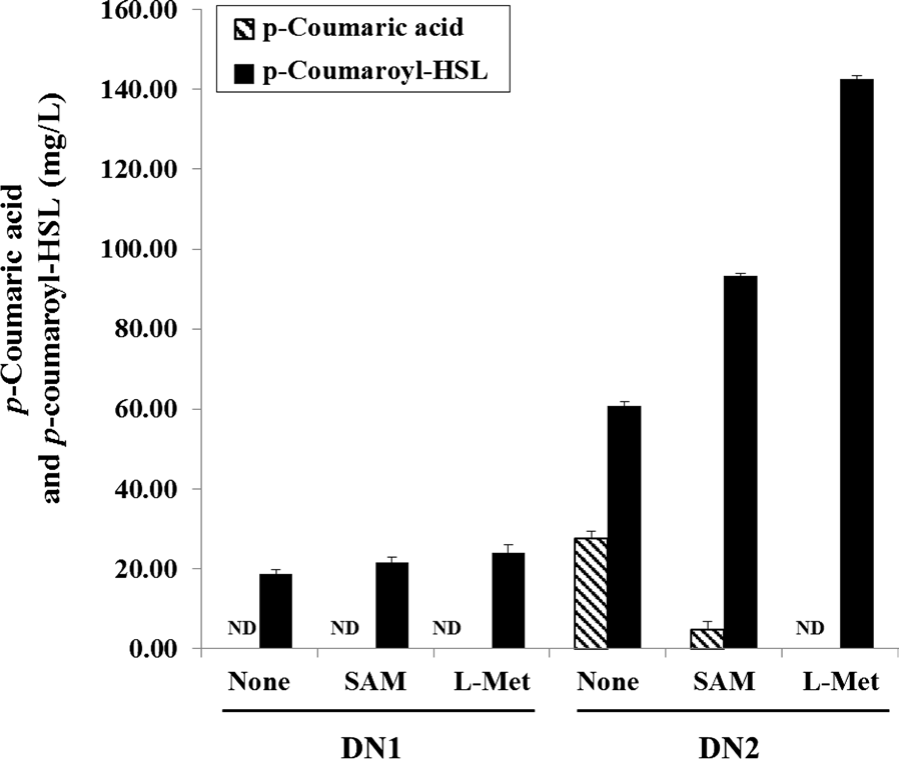
Effects of SAM or l-methionine addition on the *p*-coumaroyl-HSL production. The data were obtained after 25 h fermentation with the addition of 1 mM SAM or 1 mM l-methionine on the SM media of DN1 and DN2 strains, respectively. *Error bars* reported at one standard deviation from triplicate experiments. ND means not detected on the HPLC profile

Using the same experimental conditions described above, the tyrosine-overproducing *E. coli* ΔCOS1 strain harboring the pET-opT4R vector (DN2) produced more than 60.9 ± 0.5 mg/L of *p*-coumaroyl-HSL, an increase of 326 % over the parental strain DN1 (Fig. 6). At the same time, an expected amount of accumulated *p*-coumaric acid was also identified (Additional file 1: Figure S4). The result means that extra *p*-coumaric acid is not well converted to *p*-coumaroyl-HSL, and accumulates in the cell. Therefore, the metabolic flow to *p*-coumaroyl-HSL may be interfered by a SAM shortage during the accumulation of *p*-coumaric acid in the tyrosine-overproducing cell. Most of the HSL uses a SAM as the HSL ring donor. SAM is produced from l-methionine and ATP catalyzed by methionine adenosyltransferase (MAT) in vivo. Previous studies reported that the SAM production is improved when supplemented with excessive l-methionine in a MAT overexpressing yeast strain [28]. In order to investigate the acceleration of the metabolic flux to *p*-coumaroyl-HSL via the SAM cycle, we supplied final 1 mM of SAM and l-methionine to the culture medium of the DN1 and DN2 strains, respectively. Analysis of the product after 25 h showed that the production of the *p*-coumaroyl-HSL reached up to 93.4 ± 0.6 mg/L from the 1 mM SAM and 142.5 ± 1.0 mg/L from the 1 mM l-methionine in the DN2 strain, respectively (Fig. 6).

The production levels of *p*-coumaroyl-HSL from the tyrosine overproducing DN2 strain represented improvements of 152 and 234 % over the control culture, respectively. On the other hand, the fed SAM and l-methionine did not affect the production of *p*-coumaroyl-HSL in the DN1 strain (Fig. 6). The maximum production of *p*-coumaroyl-HSL from the DN1 strain was 21.7 ± 1.2 and 24.0 ± 2.1 mg/L, respectively, when 1 mM SAM or l-methionine were fed. However, when tyrosine was fed together with SAM or l-methionine to the culture medium of the DN1 strain, the production of *p*-coumaroyl-HSL was 16.8 ± 0.5 and 122.0 ± 1.0 mg/L, respectively (Additional file 1: Figure S6). These results mean that the tyrosine and methionine are limiting factors to the production of *p*-coumaric acid and *p*-coumaroyl-HSL, respectively.

We found a remarkable improvement in *p*-coumaroyl-HSL synthesis when the tyrosine overproducing DN2 strain was grown in l-methionine containing media, and the DN1 strain was grown in the l-methionine and tyrosine containing media. These results indicate that the elevated l-methionine assimilation pathway allows for the metabolic flux improvement of extra *p*-coumaric acid, originating from tyrosine, converting it to *p*-coumaroyl-HSL. Therefore the best metabolic engineered strains for *p*-coumaroyl-HSL production would need to activate the SAM overproducing pathway, such as enhancement of the SAM synthase gene (*metK*) activity [28, 29]. The balanced metabolic flux of tyrosine and the SAM overproducing pathway could increase intracellular *p*-coumaroyl-HSL in the heterologous host.

## Conclusions

The system in this study successfully demonstrated the *de novo* synthesis of a quorum sensing molecule, *p*-coumaroyl-HSL, using an artificial biosynthetic pathway in the heterologous host, *E. coli*. Further, the production of *p*-coumaroyl-HSL from a tyrosine overproducing strain (DN2) by feeding of SAM and l-methionine was determined to be 422 % and 650 % over the *E. coli* DN1, respectively. The titers of the *p*-coumaroyl-HSL reached up to 93.4 ± 0.6 mg/L from 1 mM SAM, and 142.5 ± 1.0 mg/L from 1 mM l-methionine, respectively, after 25 h of culturing in a glucose containing minimal medium. In addition, we demonstrated the bioconversion production of phenylacetyl-HSL analogs including cinnamoyl-HSL, *p*-coumaroyl-HSL, caffeoyl-HSL, and feruloyl-HSL in *E. coli*.

## Methods

### Chemicals

Cinnamic acid, *p*-coumaric acid, caffeic acid, ferulic acid, and *N*-(*p*-Coumaroyl)-l-homoserine lactone (*p*-coumaroyl-HSL) were purchased from Sigma-Aldrich (USA) as substrates for feeding experiments and as standards for compound identification by HPLC. Also, adenosine triphosphate (ATP), Coenzyme A (CoA), l-methionine and S-adenosyl methionine (SAM) were purchased from Sigma-Aldrich for assay of enzymes activity.

### DNA manipulation

The restriction enzymes (NEB; Takara), a *nPfu*-*Forte* DNA polymerase (Enzynomics, Korea), an ligation mix (Takara), were used according to the instructions provided by the manufacturers. The codon optimized tyrosine ammonia lyase gene (*tal*) from *Saccharothrix espanaensis* was synthesized by DNA 2.0, previously [30]. Codon optimization and synthesis of the *p*-coumaroyl CoA ligase *4CL2* gene (*4cl2nt*) from *Nicotiana tabacum* (GenBank U50846.1) was performed with the GeneGPS™ program (DNA2.0). Also, the HSL synthase gene *rpaI* from *Rhodopseudomonas palustris* (GenBank BX572593.1) were codon optimized and synthesized by Bioneer (Korea). The synthesized sequences are described in the supporting information.

### Expression and purification of 4CL2nt and RpaI proteins

*E. coli* C41(DE3) [31] containing *4cl2nt* and *rpaI* gene was grown overnight (37 °C) in 5 ml LB medium containing 50 μg/ml kanamycin, respectively. The broth of the cultures (5 mL) was used to inoculate 1 L flasks containing 300 mL of LB medium. The expressed proteins in the supernatant were purified by affinity chromatography using a Ni–NTA bead column (QIAGEN) according to the instructions provided by the manufacturers. After elution with 250 mM imidazole buffer, the solutions containing the 4CL2nt and RpaI, respectively, were dialyzed against a Tris–HCl buffer (50 mM, pH 7.4) containing 0.1 mM EDTA, 0.1 mM DTT and 10 % glycerol. Typically, the enzymes purity were >90 %, as determined by sodium dodecyl sulfate–polyacrylamide gel electrophoresis stained with Coomassie blue (Additional file 1: Figure S5).

### Assay of RpaI activity

Reaction mixtures (500 μL) containing Tris–HCl (50 mM, pH 7.4), MgCl_2_ (10 mM), ATP (1 mM), CoA (0.2 mM), SAM (0.1 mM) and each of phenolic acids (cinnamic acid, *p*-coumaric acid, caffeic acid and ferulic acid; 0.6 mM) with/without both 4CL2nt (5 μM) and RpaI (5 μM) were incubated at 30 °C for 1 h. The reaction mixtures were extracted with an equal volume of ethyl acetate. The ethyl acetate was dried and resuspended in 100 μL of methanol. The Twenty microliters of samples were applied to a J’sphere ODS-H80 column (4.6 × 150 mm i.d., 5 μm; YMC, Japan) using a high-performance liquid chromatography (HPLC) system [CH_3_CN-H_2_O (0.05 % trifluoroacetic acid), 10–100 % acetonitrile (CH_3_CN) for 25 min at flow rate of 1 mL/min; Dionex, USA] equipped with a photodiode array detector. A liquid chromatography -mass spectrometry (LC–MS) was performed using an LTQ XL linear ion trap (Thermo Scientific, USA) equipped with an electrospray ionization (ESI) source that was coupled to a rapid separation LC (RSLC; ultimate 3000, Thermo Scientific) system (ESI-LC–MS) using a HSS T3 column (Waters, UK) (2.1 × 150 mm; 2.5 µm particle size) with a linear gradient of the binary solvent system under the same HPLC conditions as described above. The data-dependent mass spectrometry experiments were controlled using the menu driven software provide with the Xcalibur system (version 2.2 SP1.48; Thermo Scientific). The compounds were identified through comparisons with the standard compounds using the observed retention time, ultraviolet spectra, and mass chromatogram. The conversion rate was calculated based on a quantitative comparison with the peak areas of absorbance at 300 nm of remained substrates and the products after enzyme reactions.

### Construction of bioconversion vectors (pET-4R) and *de novo* synthesis vector (pET-opT4R)

In order to construct an expression vector containing the *4cl2nt* and *rpaI* genes, the two genes were independently cloned into the *Nde*I and *Xho*I sites on pET-28a(+), which resulted in pET-his4CL2nt and pET-opRpaI, respectively. In order to assemble the pET-4R vector, the *4cl2nt* coding regions was amplified using the pET-4CL2nt as a template with the primers Npac (the sequence is located upstream of the T7 promoter region of the pET vector and contains the designed *Pac*I site: TTAATTAATCGCCGCGACAATTTGCGACGG) and Cspe (the sequence is located downstream of the T7 terminator region of the pET vector and contains the designed *Spe*I site: ACTAGTTCCTCCTTTCAGCAAAAAACCCCTC). The *rpaI* coding regions was amplified using the pET-opRpaI as a template with the Nspe (the sequence is located upstream of the T7 promoter region of the pET vector and contains the designed *Spe*I site: ACTAGTAGGTTGAGGCCGTTGAGCACCGCC) and Cpac (the sequence is located downstream of the T7 terminator region of the pET vector and contains the designed *Pac*I site: TTAATTAATGCGCCGCTACAGGGCGCGTCC) primers. Two amplified fragments containing the *4cl2nt* and *rpaI* coding regions, respectively, were digested with corresponding sites and cloned into pET-28a(+) using *Nde*I, *Spe*I and *Xho*I sites, which resulted in pET-4R (Table 1; Additional file 1: Figure S3). In order to construct an expression vector containing the *tal*, *4cl2nt* and *rpaI* genes, the *tal* gene were also cloned into the *Nde*I and *Xho*I sites on pET-28a(+), which resulted in pET-opTAL [12]. In order to assemble the pET-opT4R vector, the *tal* coding region was amplified using the pET-opTAL as a template with the primers opTAL-F (5′-CATATGACCCAGGTGGTTGAACGCC-3′) and Cpac. As constructed above, three amplified fragments containing the *tal*, *4cl2nt* and *rpaI* coding regions, respectively, were digested with corresponding sites and cloned into pET-28a(+) using *Nde*I, *Pac*I, *Spe*I and *Xho*I sites, which resulted in pET-opT4R (Table 1; Additional file 1: Figure S3). The gene sequences and orientations were verified via sequencing after each round of cloning, and the recombinant plasmids were transformed into *E. coli* for gene expression.

### Production of phenylacetyl-HSL analogs by *E. coli*

Recombinant *E. coli* strain (CB1) with the *rpaI* and *4cl2nt* genes (pET-4R) 37 °C in a *Luria*–*Bertani* (LB) medium containing 50 μg/mL kanamycin. The overnight culture was inoculated (1.5 %) into a fresh LB medium supplemented with the same concentration of kanamycin. The culture was grown at 37 °C to an optical density of 600 nm (OD 600) of 0.6. Then, IPTG was added to the final concentration of 1 mM, and the culture was incubated for 6 h. The cells were harvested by centrifugation, suspended, and incubated at 26 °C until 36 h in a modified synthetic medium (SM; 3 g/L KH_2_PO_4_, 7.3 g/L K_2_HPO_4_, 8.4 g/L MOPS, 2 g/L NH_4_Cl, 0.5 g/L NaCl, 0.1 ml/L Trace elements, 5 g/L (NH_4_)_2_SO_4_, 5 g/L MgSO_4_, and supplemented with 15 g/L glucose, 1 mM IPTG and 50 μg/mL kanamycin) [27, 32]. For the bioconversion experiments, the cultures were supplemented with cinnamic acid, *p*-coumaric acid, caffeic acid and ferulic acid (final concentration: 30 mg/L), respectively. The samples were collected after 24 h and analyzed by HPLC. For the detection of cinnamoyl-HSL, *p*-coumaroyl-HSL, caffeoyl-HSL and feruloyl-HSL, 1 mL of cell-free culture supernatants were filtered through 0.2 μm cellulose membrane syringe filters (Sartorius) and twenty microliters of samples were applied to a SunFire™ C18 column (250 × 4.6 mm, 5 μm; Waters, USA) using a HPLC system [CH_3_CN-H_2_O (0.05 % trifluoroacetic acid), 10–60 % acetonitrile (CH_3_CN) for 25 min at flow rate of 1 mL/min; Dionex, USA] equipped with a photodiode array detector. Further, the recombinant *E. coli* strains (DN1) and tyrosine overproducing strain (DN2) that harbored the pET-opT4R plasmid were cultured via the same method as described bioconversion. For the SAM, l-methionine and tyrosine feeding experiments, the compounds were added at concentration of 1 mM, respectively, to the fermentative media after the IPTG induction period. The detection and quantification of *p*-coumaroyl-HSL in *E. coli* was carried out as above described.

### Purification and structural elucidation of the phenylacetyl-HSL analogs

Further, the recombinant *E. coli* strains that harbored the pET-4R plasmid (DN1 and DN2) were cultured via the same method as described earlier with caffeic acid or ferulic acid, the culture volume and time were increased to 2 L for 60 h. When we supplemented with caffeic acid (10 mg/L) and ferulic acid (15 mg/L), respectively, the EtOAc-soluble material was further purified by reverse-phase HPLC (Waters Co., USA) using the YMC J’sphere ODS-H80 (10 × 250 mm, 3 mL/min) with a linear gradient from 20 to 100 % CH_3_CN containing 0.05 % TFA in order to yield caffeoyl-HSL (1.2 mg) and feruloyl-HSL (3.6 mg). The *E. coli* strain that harbored the pET-opT4R plasmid was cultured via the same method as described earlier, but the culture volume and time were increased to 2.4 L until 60 h. The *p*-coumaroyl-HSL was purified 22.8 mg. The structural elucidation of the purified compounds was undertaken using ^1^H and ^13^C NMR spectroscopy. The NMR experiments were performed on a Bruker AVANCE spectrometer (700, 900 MHz; Bruker Inc., USA). The structure of *p*-coumaroyl-HSL was determined based on the ^1^H NMR data with the values reported in the literature [7]; also caffeoyl-HSL and feruloyl-HSL were determined based on the 1D, 2D NMR data (Additional file 1: Table S1).

## Additional file

10.1186/s12934-015-0379-1 Further details of relevance to this study.
