# Supplementary material for: Production of phenylacetyl-homoserine lactone analogs by artificial biosynthetic pathway in Escherichia coli
Source: Microb Cell Fact. 2015 Nov 25;14:191. doi: 10.1186/s12934-015-0379-1 (PMC4659178; doi:10.1186/s12934-015-0379-1)
Supplement: Supplementary file 1 — 10.1186/s12934-015-0379-1 Further details of relevance to this study. [file 12934_2015_379_MOESM1_ESM.pdf]

Additional File 1: Further details of relevance to this study

Production of Phenylacetyl-Homoserine Lactone Analogs by Artificial Biosynthetic Pathway in *Escherichia coli*

Sun-Young Kang[1,2], Jae Kyoung Lee[1,2], Jae-Hyuk Jang[1], Bang Yeon Hwang[2], Young-Soo Hong[1 §]

[1]Chemical Biology Research Center, Korea Research Institute of Bioscience and Biotechnology, 30 Yeongudanji-ro, Ochang-eup, Chungbuk 363-883, Republic of Korea

[2]Department of Pharmacy Graduate School, Chungbuk National University, Cheongju 361-763, Republic of Korea

Figure S1. UV/Vis spectra of the compounds with the indicated HPLC peaks.

(a) cinnamoyl-HSL; (b) *p*-coumaroyl-HSL; (c) caffeoyl-HSL; (d) feruloyl-HSL.

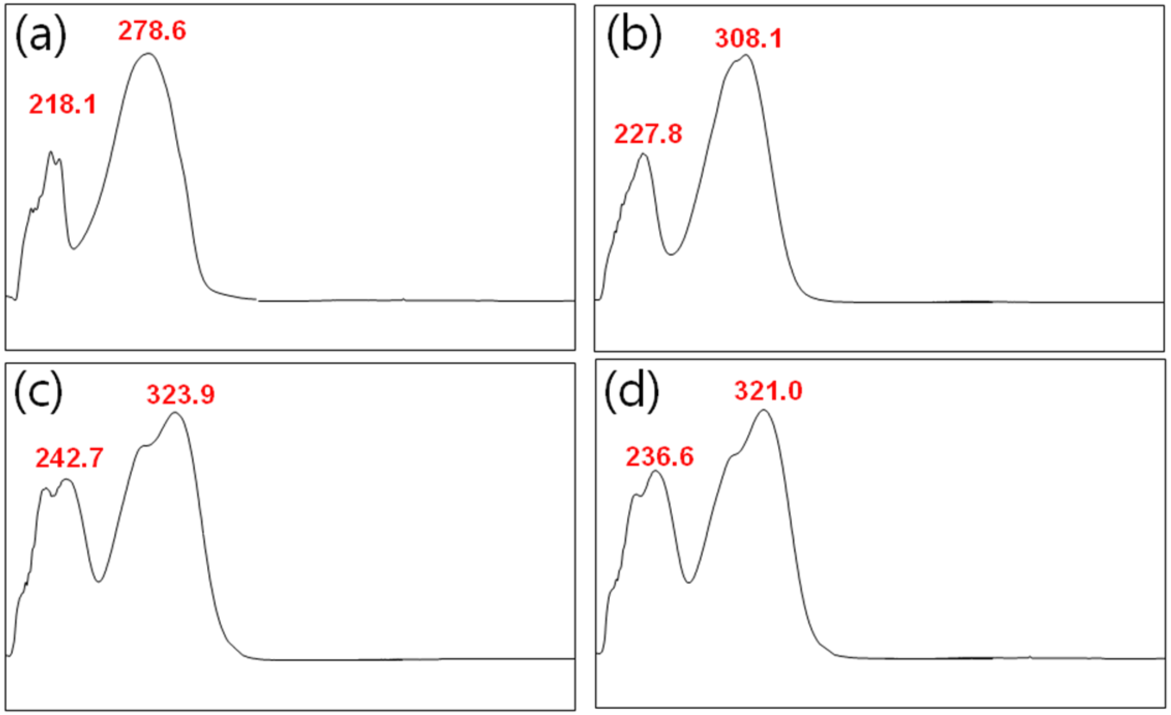

Figure S2. Structures and MS/MS spectra of phenylacetyl-HSL analogs produced by enzymatic reactions.

A) cinnamoyl-HSL; B) *p*-coumaroyl-HSL; C) caffeoyl-HSL; D) feruloyl-HSL.

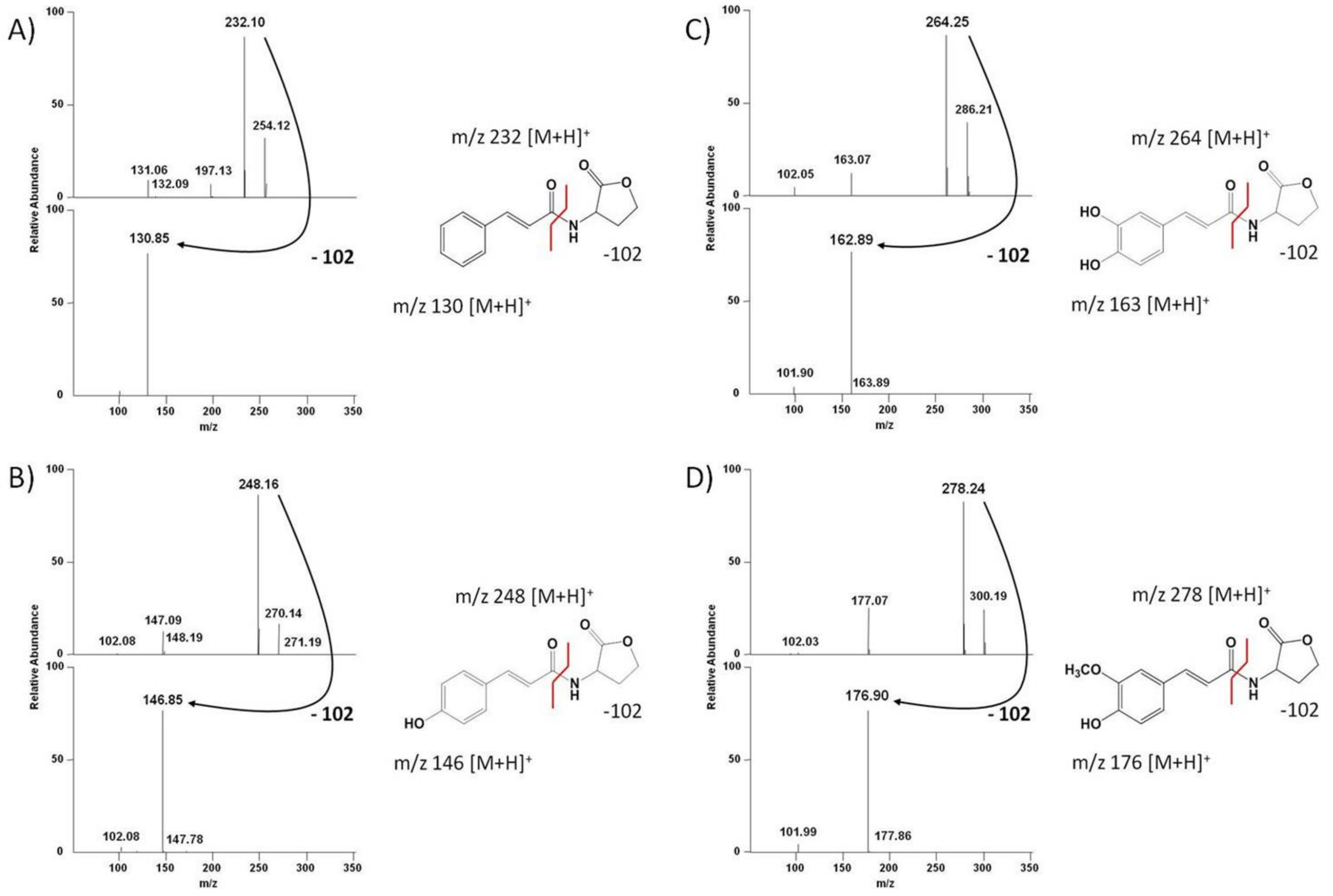

Figure S3. Organization of the gene expression vector (pET-opRpaI and pET-4CL2nt), bioconversion vector (pET-4R) and *p*-coumaroyl-HSL artificial biosynthesis vector (pET-opT4R).

Each gene contained the T7 promoter, RBS, and T7 terminator. N, *Nde*I; H, *Hind*III; P, *Pac*I; S, *Spe*I; X, *Xho*I. Codon-optimized tyrosine ammonia lyase gene *tal* from *Saccharothrix espanaensis*, codon-optimized LuxI-type synthase gene *rpaI* from *Rhodopseudomonas palustris*, *p*-coumaroyl-CoA ligase gene *4cl2nt* from *Nicotiana tabacum*,

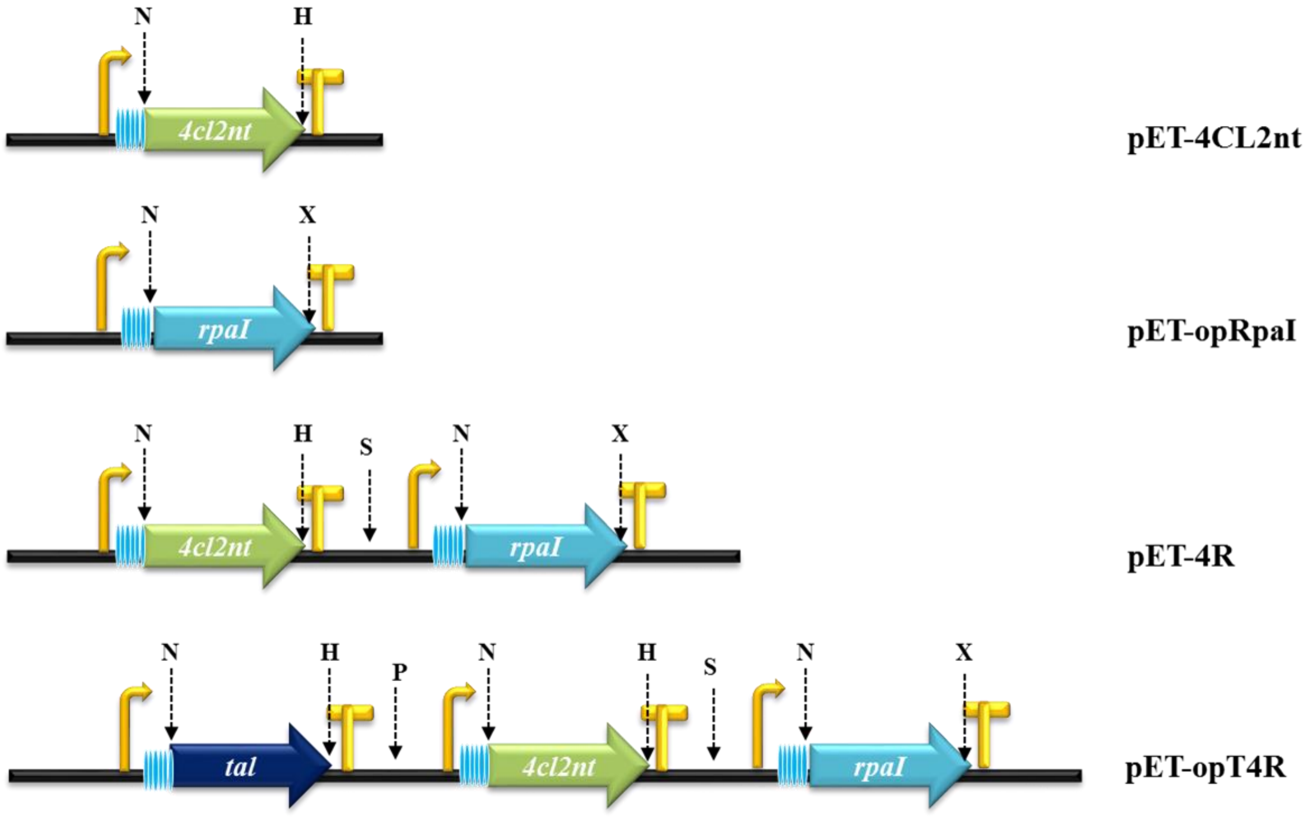

Figure S4. HPLC profile of the 25 hours culture broth of tyrosine overproducing *E. coli* (ΔCOS1) harboring pET-opT4R (DN2).

Peak 2, *p*-coumaric acid; peak 6, *p*-coumaroyl-HSL.

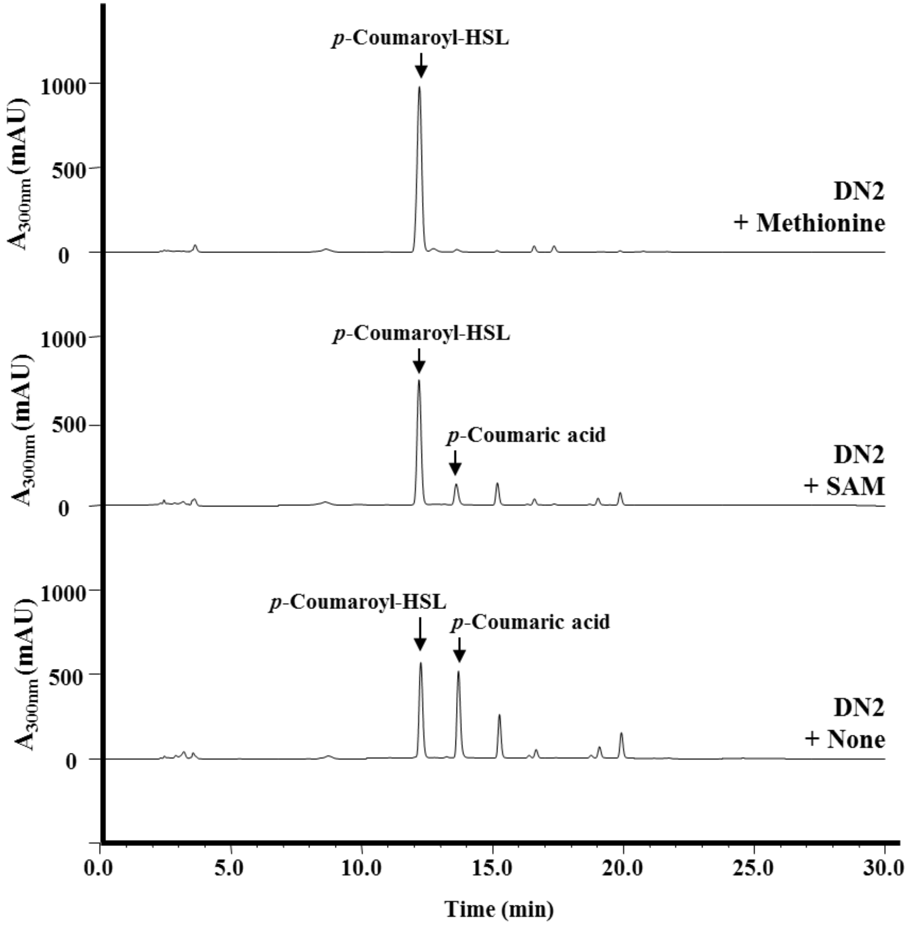

Figure S5. SDS-PAGE analysis of purified His-tagged 4CL2nt and RpaI enzymes.

M, size markers; 4CL2nt (A, 61.5kDa) and RpaI (B, 26.7kDa) after affinity chromatography with His-binding resin, respectively.

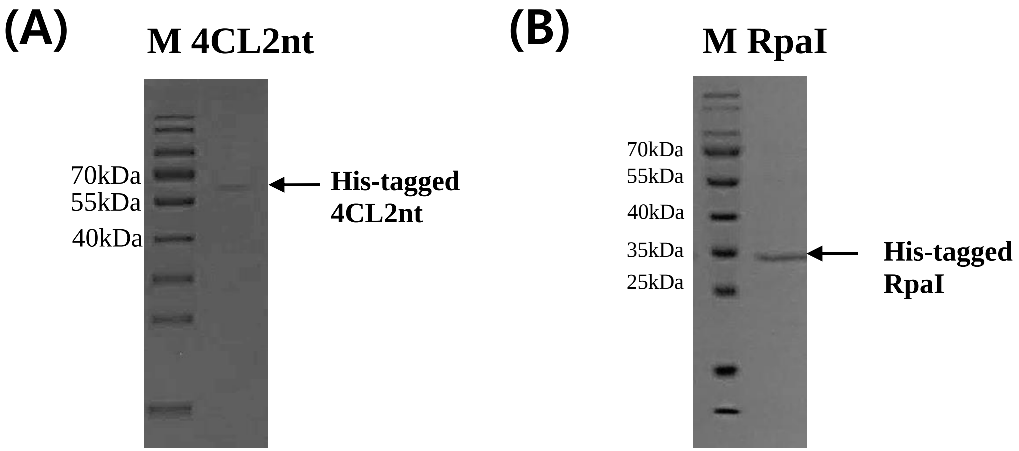

Figure S6. Effects of SAM or L-methionine with combinatorial addition of tyrosine on the *p*-coumaroyl-HSL production in the DN1 strain.

The data were obtained after 25 h fermentation with the addition of 1 mM SAM or 1 mM L-methionine with 1 mM tyrosine on the SM media of the DN1 strain, respectively. The addition of tyrosine only (Tyr) or 1 mM SAM + Tyrosine (Tyr+SAM) or 1 mM L-methionine + Tyrosine (Tyr+Met) on the glucose media (SM) of the DN1 strain, respectively. Error bars reported at one standard deviation from triplicate experiments.

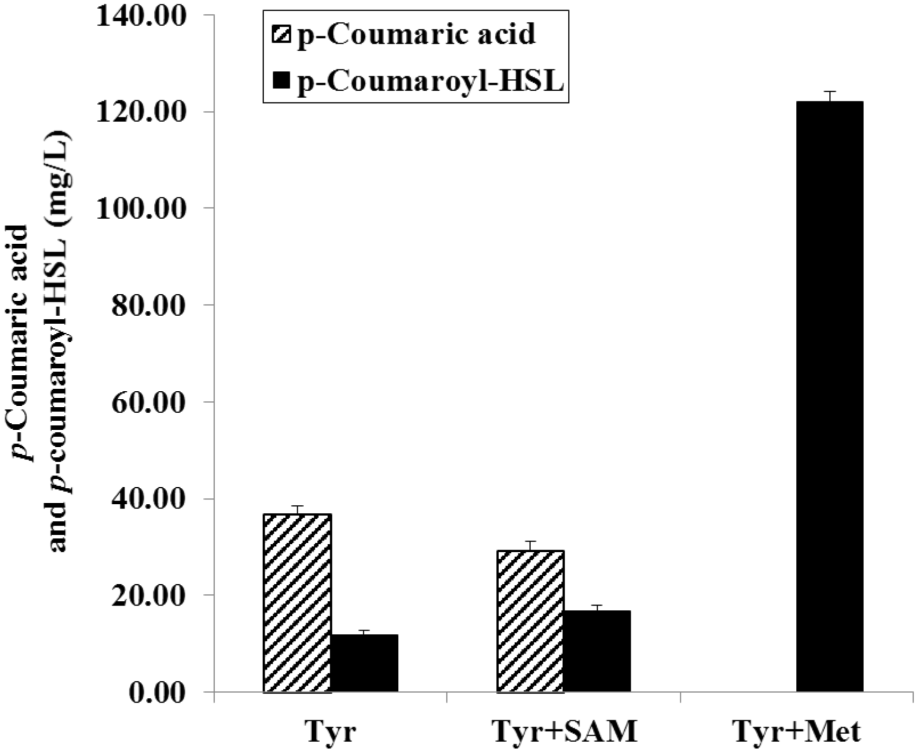

Figure S7. ^{1}H and ^{13}C NMR spectrum of phenylacetyl-HSL analogs in DMSO-*d6*. A) *p*-coumaroyl HSL, B) caffeoyl-HSL and C) feruloyl-HSL

A)

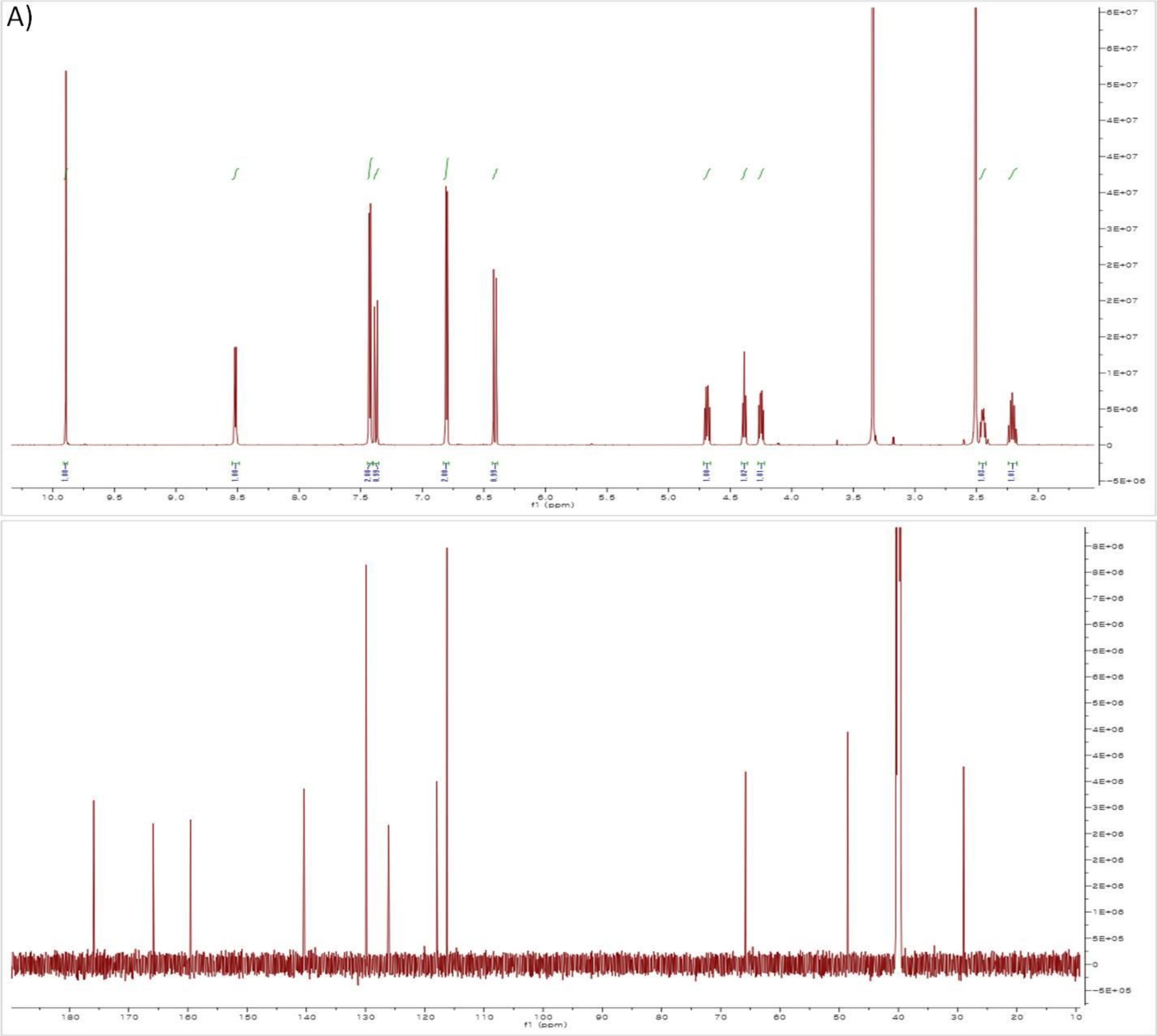
10.0 9.5 9.0 8.5 8.0 7.5 7.0 6.5 6.0 5.5 5.0 4.5 4.0 3.5 3.0 2.5 2.0
f1 (ppm)
180 170 160 150 140 130 120 110 100 90 80 70 60 50 40 30 20 10
f1 (ppm)

B)

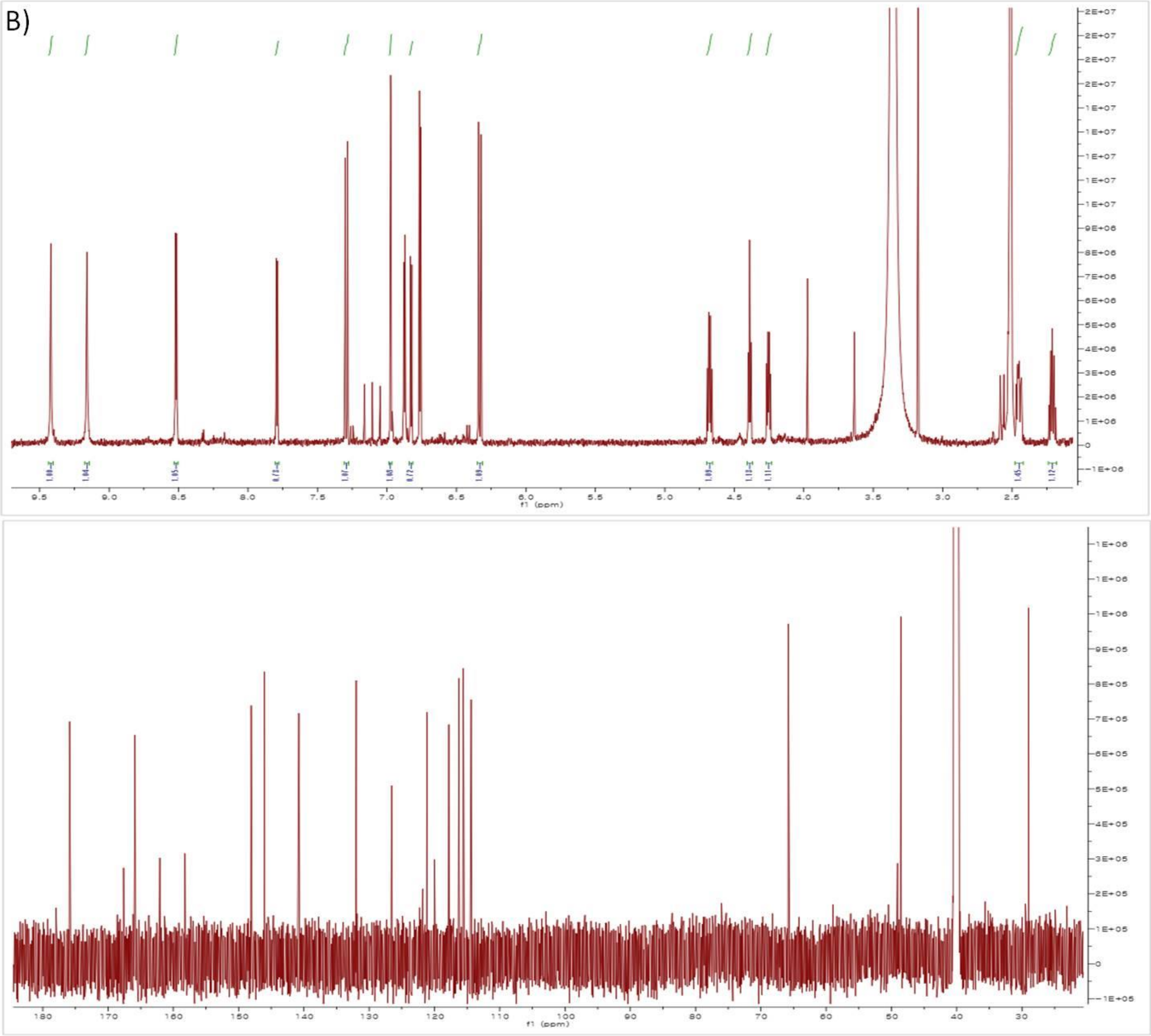

C)

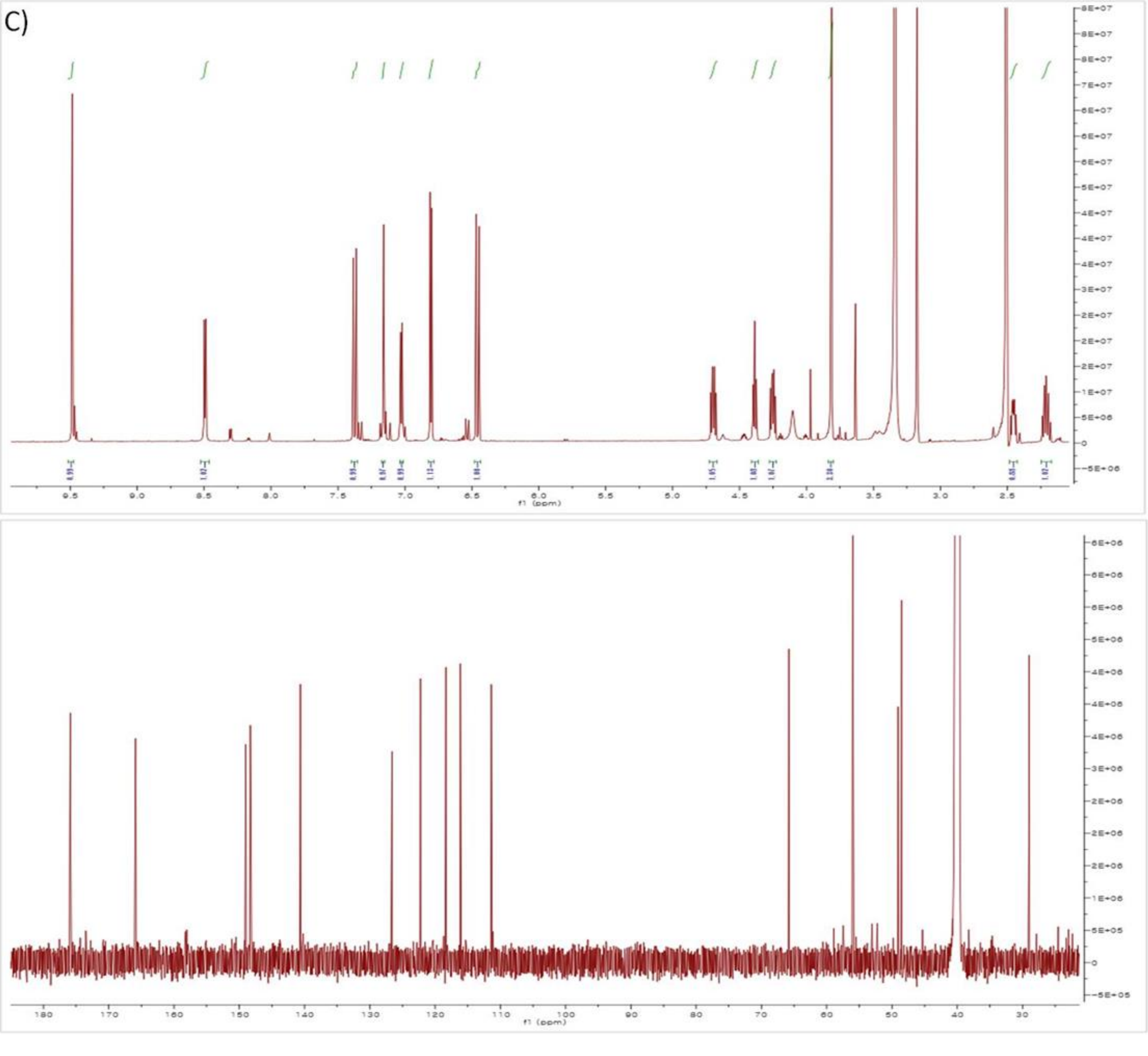

Table S1. ^{1}H and ^{13}C NMR data of phenylacetyl-HSL analogs

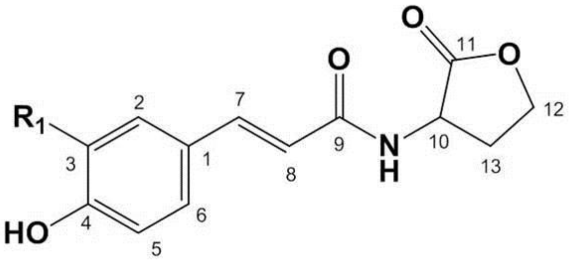

p-Coumaroyl HSL : R = H
Caffeoyl HSL : R = OH
Feruloyl HSL : R = OCH_3

	p-Coumaroyl HSL		Caffeoyl HSL		Feruloyl HSL	
Position	δc^b (176MHZ)	$\delta_H{}^a$ (J in Hz) (700MHZ)	δc^b (176MHZ)	$\delta_H{}^a$ (J in Hz) (900MHZ)	δc^b (176MHZ)	$\delta_H{}^a$ (J in Hz) (700MHZ)
1	126.6		126.6		126.6	
2	129.8	7.43 (d, J = 8.6 Hz, 1H)	114.3	6.97 (d, J = 1.8 Hz, 1H)	111.3	7.16 (d, J = 1.9 Hz, 1H)
3	116.2	6.81 (d, J = 8.6 Hz, 1H)	148.0		149.0	
4	159.5		145.9		148.3	
5	116.2	6.81 (d, J = 8.6 Hz, 1H)	115.5	6.83 (d, J = 8.6 Hz, 1H)	122.2	7.03 (dd, J = 8.2, 1.9 Hz, 1H)
6	129.8	7.43 (d, J = 8.6 Hz, 1H)	131.9	7.79 (d, J = 8.6 Hz, 1H)	116.1	6.81 (d, J = 8.1 Hz, 1H)
7	140.4	7.38 (d, J = 15.7 Hz, 1H)	140.7	7.29 (d, J = 15.7 Hz, 1H)	140.7	7.38 (d, J = 15.7 Hz, 1H)
8	117.9	6.41 (d, J = 15.7 Hz, 1H	117.7	6.33 (d, J = 15.7 Hz, 1H)	118.4	6.46 (d, J = 15.7 Hz, 1H)
9	165.8		165.8		165.8	
10	48.5	4.68 (m, 1H)	48.3	4.68 (dd, J = 18.5, 9.5 Hz, 1H)	48.3	4.70 (dt, J = 10.9, 8.9 Hz, 1H)
11	175.9		175.9		175.8	
12	65.8	4.39 (m, 1H), 4.25(m, 1H)	65.7	4.39 (m, 1H), 4.25 (m, 1H)	65.8	4.39 (m, 1H). 4.24 (m, 1H)
13	28.9	2.45 (m, 1H), 2.21 (m, 1H)	28.8	2.48 (m, 1H), 2.21 (m, 1H)	29.0	2.48 (m, 1H), 2.21(m, 1H)
		8.52 (d, J = 8.0 Hz, 1H - NH)		8.52 (d, J = 8.0 Hz, 1H - NH)	56.0	3.81 (s, 3H)
		9.89 (s, 1H - 4-OH)		9.16 (s, 1H - 3-OH)		8.49 (d, J = 8.0 Hz, 1H - NH)
				9.42 (s, 1H - 4-OH)		9.49 (s, 1H - 4-OH)

Table S2. Synthesized sequences.

tal	ATGACCCAGGTGGTTGAACGCCAGGCCGATCGCCTGAGTAGTCGTGAATACTTAGCTCGCGTCGTTCGTAGTGCCGGCTGGGATGCGGGCCTAACCTCTTGTAC<br>AGATGAAGAAATTGTTCGCATGGGCGCGTCAGCCCGCACCATCGAGGAATATTTAAAAAGTGATAAACCGATTTATGGTTTAACCCAAGGCTTCGGCCCGCTGG<br>TACTGTTTGATGCGGATAGCGAATTAGAACAGGGTGGTAGCCTGATTAGCCATCTGGGCACCGGTCAGGGCGCGCCGCTGGCGCCGGAAGTGAGTCGTTTAATT<br>CTGTGGCTGCGTATTCAAAACATGCGCAAAGGTTATAGCGCCGTTAGCCCGGTTTTCTGGCAAAAACTGGCAGACCTATGGAATAAAGGCTTTACCCCGGCAAT<br>TCCGCGTCATGGTACCGTTTCCGCCTCGGGTGATCTGCAACCGCTGGCGCATGCCGCGCTGGCATTCACCGGCGTGGGTGAAGCGTGGACCCGCGATGCAGAT<br>GGCCGCTGGAGCACCGTTCCGGCTGTTGATGCCCTGGCAGCGCTGGGTGCCGAACCGTTTGATTGGCCTGTCCGCGAAGCACTGGCGTTTGTTAATGGCACCG<br>GAGCCAGCCTGGCGGTTGCTGTTTTAAATCATCGTTCTGCCCTGCGCCTGGTTCGCGCGTGTGCGGTACTGAGCGCACGCCTGGCGACCCTGCTGGGCGCAAA<br>TCCGGAACATTATGACGTTGGTCATGGCGTTGCCCGCGGTCAGGTTGGCCAGCTGACCGCGGCGGAATGGATTCGTCAGGGCCTGCCACGTGGTATGGTGCGC<br>GATGGAAGCCGTCCGTTGCAGGAACCTTATAGCCTTCGCTGCGCTCCGCAGGTTCTAGGCGCTGTTCTGGATCAGCTGGACGGTGCGGGTGACGTGCTGGCCC<br>GCGAAGTTGATGGTTGCCAGGATAACCCTATTACCTACGAAGGTGAATTGCTGCATGGCGGTAACTTCCATGCCATGCCGGTTGGTTTTGCAAGTGATCAGATTG<br>GTCTGGCGATGCACATGGCGGCCTACCTGGCTGAACGCCAGCTGGGCCTGCTGGTTAGCCCGGTAACCAATGGTGATTTACCACCGATGCTGACCCCGCGTGC<br>CGGCCGTGGTGCGGGTCTTGCTGGCGTCCAGATTTCTGCCACCAGCTTCGTTTCTCGTATTCGCCAACTGGTTTTCCCGGCGTCTCTGACCACCCTGCCGACCA<br>ACGGTTGGAATCAAGACCATGTACCGATGGCACTGAATGGCGCTAATAGCGTTTTCGAAGCACTGGAACTGGGTTGGTTAACCGTTGGAAGCCTGGCGGTGGG<br>CGTTGCACAGCTGGCGGCGATGACCGGTCATGCGGCTGAAGGGGTTTGGGCAGAACTGGCAGGCATTTGCCCGCCGTTAGATGCCGACCGTCCGCTGGGTGC<br>GGAAGTTCGCGCAGCCCGTGATCTGCTGAGCGCGCACGCTGATCAGCTGTTGGTGGACGAAGCCGATGGTAAAGACTTTGGCTAA
4cl2nt	ATGGAGAAAGACACGAAGCAAGTTGACATCATTTTTCGCTCGAAACTGCCGGACATTTACATTCCGAATCATCTGCCGCTGCATAGCTACTGCTTCGAGAACAT<br>TTCTGAATTTTCTAGCCGTCCGTGTCTGATTAACGGTGCCAATAAACAGATCTATACGTACGCGGACGTCGAGTTGAACAGCCGTAAGGTCGCAGCGGGTCTGC<br>ACAAGCAAGGCATCCAGCCTAAAGATACCATCATGATTCTGTTGCCAAATTCTCCGGAGTTTGTGTTTGCGTTTATCGGCGCAAGCTACCTGGGTGCGATTAGCA<br>CGATGGCAAATCCGCTGTTTACCCCGGCTGAGGTTGTTAAACAAGCAAAAGCCAGCAGCGCGAAGATCATCGTGACCCAAGCATGCCACGTCAACAAAGTTA<br>AGGACTATGCCTTCGAAAATGACGTCAAGATCATTTGCATCGATAGCGCGCCTGAAGGTTGTCTGCATTTCAGCGTTCTGACGCAGGCTAACGAACACGATATT<br>CCGGAAGTTGAGATTCAGCCGGACGATGTGGTGGCCCTGCCGTACTCCAGCGGTACCACCGGCCTGCCGAAAGGCGTTATGCTGACCCACAAGGGCCTGGTG<br>ACGAGCGTCGCCCAGCAGGTCGATGGTGAAAACCCGAACCTGTACATCCACAGCGAAGATGTTATGCTGTGTGTTCTGCCACTGTTCCACATCTATTCCCTGAA<br>CAGCGTCCTGCTGTGCGGCCTGCGTGTGGGCGCTGCCATTTTGATTATGCAGAAGTTTGACATTGTCAGCTTCTTGGAACTGATCCAACGCTACAAGGTGACGA<br>TCGGTCCGTTCGTCCCGCCGATTGTTTTGGCCATTGCAAAAAGCCCAATGGTGGATGACTATGACCTGTCGAGCGTGCGTACCGTGATGTCCGGTGCAGCGCCG<br>CTGGGCAAAGAGCTGGAGGATACCGTTCGTGCGAAGTTTCCGAATGCGAAACTGGGTCAAGGCTACGGTATGACTGAAGCAGGTCCGGTGCTGGCGATGTGC<br>TTGGCGTTCGCGAAAGAGCCGTTCGAAATCAAAAGCGGTGCGTGCGGTACCGTGGTGCGTAATGCTGAAATGAAAATTGTGGATCCGAAAACCGGCAACAGC<br>CTGCCGCGCAACCAGAGCGGTGAGATTTGTATTCGCGGTGACCAGATTATGAAGGGCTACCTGAATGACCCGGAGGCCACTGCGCGTACGATCGACAAAGAGG<br>GTTGGCTGTATACCGGCGACATCGGTTATATCGATGACGACGACGAGCTGTTCATCGTTGATCGCCTGAAAGAGTTGATTAAGTACAAGGGTTTCCAAGTTGCG<br>CCTGCGGAACTGGAGGCTCTGCTGTTGAATCATCCGAACATTAGCGATGCAGCAGTCGTTCCGATGAAGGATGAGCAGGCGGGTGAAGTTCCGGTCGCGTTTG<br>TTGTGCGTAGCAACGGCAGCACGATCACCGAGGATGAGGTAAAGGATTTCATTTCCAAACAAGTCATCTTCTATAAGCGTATCAAGCGTGTGTTTTTCGTCGAT<br>GCAATCCCGAAAAGCCCGTCCGGTAAGATCCTGCGCAAAGACTTGCGTGCGAAGCTGGCGGCAGGTCTGCCGAATTAGTAA

rpaI	ATGCAAGTTCATGTTATTCGTCGCGAGAACCGCGCGCTCTATGCCGGTTTGCTAGAAAAATATTTCCGTATTCGTCACCAAATCTACGTCGTAGAACGCGGCTGG<br>AAAGAATTGGATCGGCCGGATGGACGAGAAATTGATCAGTTCGATACCGAAGACGCGGTGTATCTTTTAGGTGTCGACAATGATGATATTGTAGCTGGCATGCG<br>TATGGTGCCGACCACGTCTCCGACACTTCTGAGCGATGTTTTTCCACAACTCGCGCTGGCTGGTCCAGTAAGAAGGCCTGATGCCTATGAGTTATCTCGGATATT<br>TGTGGTTCCTCGTAAGCGCGGAGAGCATGGGGGCCCACGTGCTGAGGCAGTGATACAGGCGGCCGCAATGGAATACGGTTTATCGATTGGCCTGTCAGCCTTT<br>ACTATCGTACTGGAAACTTGGTGGCTGCCGCGACTGGTTGACCAGGGCTGGAAAGCAAAACCTTTAGGTCTGCCTCAGGATATCAATGGATTTTCCACCACAG<br>CAGTCATCGTTGATGTTGACGATGATGCTTGGGTCGGTATTTGTAATAGACGCAGTGTTCCCGGACCCACGTTAGAATGGAGAGGGTTAGAAGCAATACGGCGT<br>CATAGTCTTCCGGAATTTCAGGTGATTTCATAA
